# Crystal structures of polymerized lithium chloride and dimethyl sulfoxide in the form of {2LiCl·3DMSO}_*n*_ and {LiCl·DMSO}_*n*_

**DOI:** 10.1107/S2056989022011896

**Published:** 2023-01-01

**Authors:** Nichole R. Valdez, David J. Herman, Martin B. Nemer, Mark A. Rodriguez, Eric Allcorn

**Affiliations:** a Sandia National Laboratories, 1515 Eubank Blvd. SE, Albuquerque, NM 87123, USA; University of Neuchâtel, Switzerland

**Keywords:** crystal structure, LiCl, DMSO, polymer

## Abstract

Two novel LiCl·DMSO polymer structures were created by combining dry LiCl salt with dimethyl sulfoxide (DMSO), namely, *catena*-poly[[chlorido­lithium(I)]-μ-(dimethyl sulfoxide)-κ^2^ *O*:*O*-[chlorido­lithium(I)]-di-μ-(dimethyl sulfoxide)-κ^4^ *O*:*O*], [Li_2_Cl_2_(C_2_H_6_OS)_3_]_*n*_, and *catena*-poly[lithium(I)-μ-chlorido-μ-(dimethyl sulfoxide)-κ^2^ *O*:*O*], [LiCl(C_2_H_6_OS)]_*n*_. The initial synthesized phase had very small block-shaped crystals (<0.08 mm) with monoclinic symmetry and a 2 LiCl: 3 DMSO ratio. As the solution evaporated, a second phase formed with a plate-shaped crystal morphology. After about 20 minutes, large (>0.20 mm) octa­hedron-shaped crystals formed. The plate crystals and the octa­hedron crystals are the same tetra­gonal structure with a 1 LiCl: 1 DMSO ratio. These structures are reported and compared to other known LiCl·solvent compounds.

## Abstract 2

The novel crystal structures of two LiCl·DMSO polymer phases are described.

## 1. Chemical context

Lithium salts are soluble in a wide range of solvents and are widely used in lithium-metal and lithium-ion battery applications (Bushkova *et al.*, 2017 ▸; Mauger *et al.*, 2018 ▸; Younesi *et al.* 2015 ▸). While typically implemented as liquid electrolyte solutions, the lithium salt and solvent systems can also form complex mol­ecular phases, including inter­calating compounds (Yamada *et al.*, 2010 ▸), crystalline solvates (Ugata *et al.*, 2021 ▸), and polymeric structures (Rao *et al.*, 1984 ▸; Chivers *et al.*, 2001 ▸).

Dimethyl sulfoxide (DMSO) and lithium chloride (LiCl) are very common materials in many industries, and have each been used in novel battery systems, including solid-polymer (Voigt & van Wüllen, 2012 ▸), dual-ion (Wang *et al.*, 2022 ▸), lithium–oxygen (Togasaki *et al.*, 2016 ▸; Reddy *et al.*, 2018 ▸; Zhang *et al.*, 2021 ▸) and molten-salt electrolyte batteries (Allcorn *et al.*, 2020 ▸). Given that DMSO and water both exhibit a coordination number of four solvent mol­ecules per cation (Megyes *et al.*, 2006 ▸; Bouazizi & Nasr, 2007 ▸), it is reasonable to hypothesize that the two solvents solvate lithium ions similarly. For Li^+^ cations in binary solvent solutions of DMSO and water, the solvent mol­ecules are analogous; there is effectively no selective solvation of Li^+^ cations for either DMSO or water (Pasgreta *et al.*, 2007 ▸). Thus it is not surprising that lithium salts would form similar crystalline phases when comparing phase diagrams in pure DMSO (Kirillov *et al.*, 2015 ▸) and pure water (Perron *et al.*, 1997 ▸). Since LiCl is hydrated with 1–2 water mol­ecules per Li^+^ cation in ambient conditions (Conde, 2004 ▸; Pátek & Klomfar, 2006 ▸), it is reasonable to expect that LiCl would form similar if not analogous solvate phases in DMSO (1–2 DMSO mol­ecules per Li^+^ cation). 
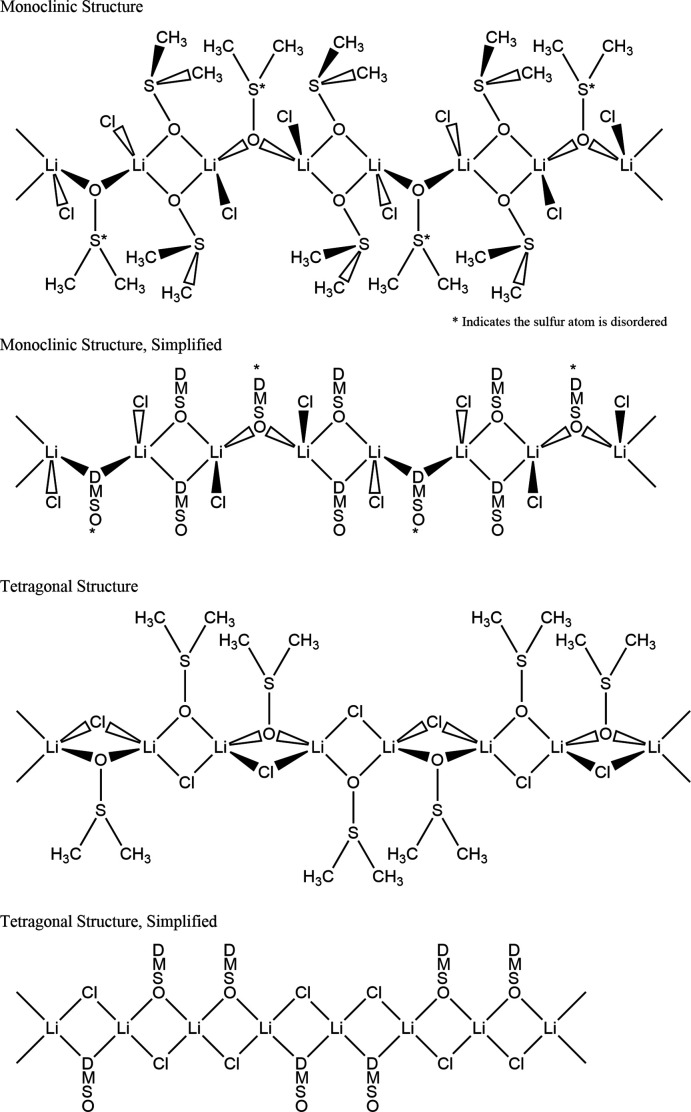


## 2. Synthesis and crystallization

Material samples were prepared using lithium chloride (Acros Organics, LiCl 99% anhydrous) and dimethyl sulfoxide (Sigma-Aldrich, C_2_H_6_OS ≥99.9% anhydrous). Before use, the LiCl was heated to 423.15 K (150°C) under vacuum to remove any trace moisture, and the sample preparation was carried out in a humidity-controlled dry room, dew point below 223.15 K (−50°C).

Dry LiCl was added to a jar of DMSO at a ratio of 5 g LiCl per 25 g DMSO, approximately twice the limit at 298.15 K (25°C) before saturation is initially observed (Xin *et al.*, 2018 ▸). As the salt tends to agglomerate quickly upon being added to the DMSO, the initial larger agglomerates were manually broken up. The jar was then sealed and the entire solution was stirred vigorously with a magnetic stir bar for 3 days. An aliquot of the sample (solids and saturated DMSO combined) was removed for analysis. During sample preparation for single crystal X-ray diffraction analysis, DMSO evaporated from the sample aliquot, resulting in a second, likely metastable, phase with different crystal morphology.

## 3. Structural Commentary

*Monoclinic Crystals*

**Figure 1:**
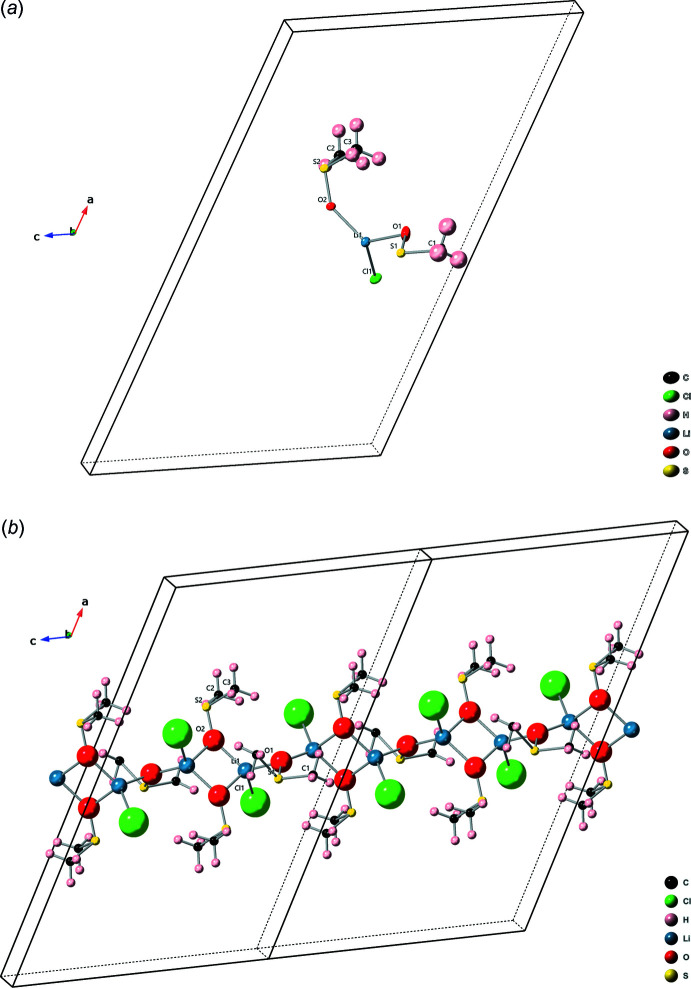
Monoclinic structure: asymmetric unit (*a*) and polymeric chain view (*b*). The repeating unit of the polymer has four LiCl and six DMSO. The sulfur atom of the DMSO is disordered across two positions. Only one position is shown.

The initial crystals synthesized as described in the previous section are small (<0.08 mm), block-shaped, and have monoclinic symmetry *C*2/*c*. The polymer has a 2 LiCl: 3 DMSO ratio, and the repeating unit is composed of four LiCl, and six DMSO, see Fig. 1 ▸ and Scheme.

**Figure 2:**
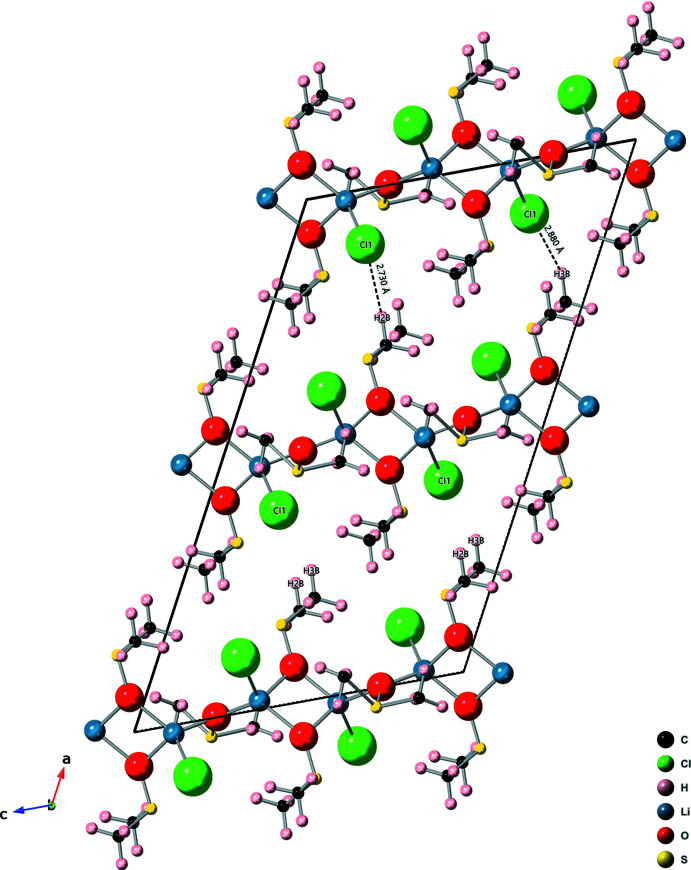
Monoclinic structure: packing diagram. The structure is held together by hydrogen bonding. The hydrogen bonds between Cl1 and H2*B* as well as Cl1 and H3*B* are shown.

The polymers appear to be held together by hydrogen bonding, see Table 1 ▸ and packing diagram Fig. 2 ▸. The most notable bond is between Cl1 and H2*B* (2.730 Å), where the Cl atom on one polymer chain is connected to one of the hydrogen atoms on one of the methyl groups of the non-disordered DMSO mol­ecule of another polymer chain. There is likely some hydrogen bond contribution from the adjacent H3*B*, which is located on the other methyl group of the same DMSO mol­ecule (hydrogen-bond length 2.88 Å). If the disordered DMSO mol­ecule contributes to hydrogen bonding between polymer chains, it would be through hydrogen H1*A* and Cl1, however this bond is very long [2.98 (3) Å]. The other values in the table represent hydrogen bonding between a DMSO mol­ecule and a Cl atom along the same polymer chain.

*Tetra­gonal Crystals*

**Figure 3:**
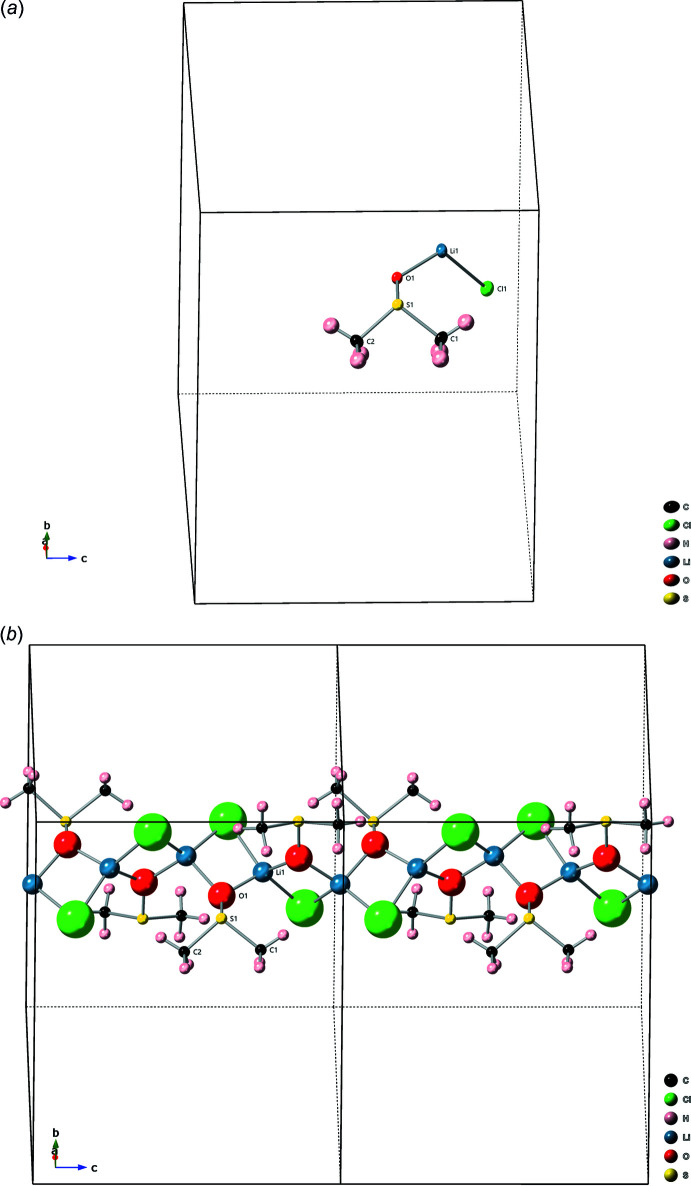
Tetra­gonal structure: asymmetric unit (*a*) and polymeric chain view (*b*). The repeating unit of the polymer has four LiCl and four DMSO. The DMSO mol­ecules in this structure are not disordered.

The second crystal phase formed during sample preparation as DMSO evaporated. At first, plate-shaped crystals appeared among the smaller block-shaped crystals. As more time passed (∼20 minutes), much larger (0.2–0.4 mm) octa­hedron-shaped crystals formed. The plate crystals and the octa­hedron crystals are the same tetra­gonal *I*4_1_/*a* structure with a 1 LiCl: 1 DMSO ratio. The repeating unit has four LiCl, and four DMSO. The DMSO mol­ecules are not disordered in this structure, see Fig. 3 ▸ and Scheme.

**Figure 4:**
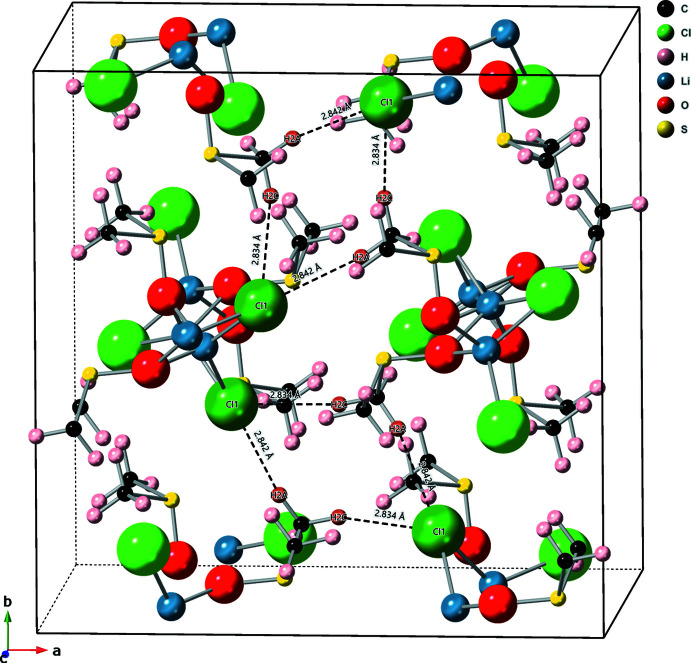
Tetra­gonal structure: packing diagram. The structure is held together by hydrogen bonding. The hydrogen bonds between Cl1 and H2*A* and Cl1 and H2*C* are shown. The atoms involved in hydrogen bonding are darkened for clarity.

As with the monoclinic structure, the tetra­gonal structure is composed of polymer chains held together by hydrogen bonding, see Table 2 ▸ and the packing diagram Fig. 4 ▸. The Cl1 of one chain is linked to the DMSO of another chain through H2*A* [2.84 (2) Å] and H2*C* [2.83 (2) Å]. There may be some contribution from H1*B*, though the bond is much longer [2.95 (2) Å].

## 4. Database Survey

After the structures were solved, a search was performed on the Cambridge Structural Database (CSD, version 5.43, November 2021; Groom *et al.*, 2016 ▸). There were only two results with the relevant chemistry, and neither had DMSO. One was a LiCl sulfolane adduct (SIWFOT; Harvey *et al.*, 1991 ▸), and the other was a crown ether complex (XEGBIX; Reuter *et al.*, 2017 ▸). These two LiCl·DMSO structures are novel, and other phases likely exist in the LiCl–DMSO system as a function of temperature, analogous to the LiCl–H_2_O system (Perron *et al.*, 1997 ▸). An extensive list of LiCl structures with various other ligands can be found in Chivers *et al.* (2001 ▸).

## 5. Refinement

Crystal data, data collection, and structure refinement details are summarized in Table 3 ▸. One of the DMSO mol­ecules on the monoclinic structure is disordered. The two positions of the sulfur atom show a *C*_2_ symmetry-related disorder about the oxygen atom in the *b*-axis direction of the unit cell. An attempt was made to model the disorder using a lower space group (*Cc*); however, the refinement was unstable. Without the ability to use a PART instruction, the DMSO mol­ecule was fixed to an occupancy of 0.5. The hydrogen atoms on the disordered DMSO mol­ecule were placed manually. For the monclinic structure, all H atoms were refined with *U*_iso_(H) = 1.5*U*_eq_(C). Bond-length restraints of 0.98 ± 0.02 Å were applied to the H atoms on C2 and C3.

## Supplementary Material

Crystal structure: contains datablock(s) Monoclinic, Tetragonal. DOI: 10.1107/S2056989022011896/tx2060sup1.cif

CCDC references: 2226319, 2226318

Additional supporting information:  crystallographic information; 3D view; checkCIF report

## Figures and Tables

**Table 1:** Hydrogen-bond geometry (Å, °) for the monoclinic structure[Chem scheme1]

*D*—H⋯*A*	*D*—H	H⋯*A*	*D*⋯*A*	*D*—H⋯*A*
C1—H1*A*⋯Cl1^i^	0.97 (1)	2.98 (4)	3.569 (3)	120 (3)
C1—H1*B*⋯Cl1^ii^	0.97 (1)	2.79 (2)	3.690 (3)	156 (3)
C2—H2*A*⋯Cl1^iii^	0.98	2.71	3.680 (3)	169
C2—H2*B*⋯Cl1^iv^	0.98	2.73	3.632 (3)	153
C3—H3*B*⋯Cl1^iv^	0.98	2.88	3.752 (3)	149

Symmetry codes: (i) 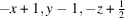 ; (ii) 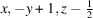 ; (iii) 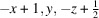 ; (iv) 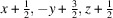 .

**Table 2:** Hydrogen-bond geometry (Å, °) for the tetragonal structure[Chem scheme1]

*D*—H⋯*A*	*D*—H	H⋯*A*	*D*⋯*A*	*D*—H⋯*A*
C1—H1*B*⋯Cl1^i^	0.98 (2)	2.95 (2)	3.821 (2)	148 (2)
C2—H2*A*⋯Cl1^ii^	0.95 (2)	2.84 (2)	3.768 (2)	165 (2)
C2—H2*C*⋯Cl1^i^	0.96 (2)	2.83 (2)	3.716 (2)	153 (2)

Symmetry codes: (i) 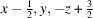 ; (ii) 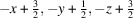 .

**Table 3:** Experimental details

	Monoclinic	Tetragonal
Crystal data		
Chemical formula	[Li_2_Cl_2_(C_2_H_6_OS)_3_]	[LiCl(C_2_H_6_OS)]
*M*_r_	319.16	120.52
Crystal system, space group	Monoclinic, *C*2/*c*	Tetragonal, *I*4_1_/*a*
Temperature (K)	100	100
*a*, *b*, *c* (Å)	19.2841 (17), 7.6436 (7), 11.5335 (10)	14.2411 (14), 14.2411 (14), 10.8809 (16)
α, β, γ (°)	90, 118.315 (5), 90	90, 90, 90
*V* (Å^3^)	1496.6 (2)	2206.7 (5)
*Z*	4	16
Radiation type	Cu *K*α	Cu *K*α
μ (mm^−1^)	7.72	8.49
Crystal size (mm)	0.07 × 0.07 × 0.05	0.4 × 0.4 × 0.4

Data collection		
Diffractometer	Bruker APEXII CCD	Bruker APEXII CCD
Absorption correction	Multi-scan (*SADABS*; Krause *et al.*, 2015 ▸)	Multi-scan (*SADABS*; Krause *et al.*, 2015 ▸)
*T*_min_, *T*_max_	0.639, 0.754	0.513, 0.754
No. of measured, independent and observed [*I* > 2σ(*I*)] reflections	10606, 1417, 1166	8174, 1072, 1030
*R*_int_	0.064	0.047
(sin θ/λ)_max_ (Å^−1^)	0.610	0.618

Refinement		
*R*[*F* ^2^ > 2σ(*F* ^2^)], *wR*(*F* ^2^), *S*	0.035, 0.081, 1.09	0.028, 0.073, 1.07
No. of reflections	1417	1072
No. of parameters	92	79
No. of restraints	68	0
H-atom treatment	H atoms treated by a mixture of independent and constrained refinement	All H-atom parameters refined
Δρ_max_, Δρ_min_ (e Å^−3^)	0.46, −0.39	0.32, −0.37

Computer programs: *APEX2* and *SAINT* (Bruker, 2016 ▸), *SHELXT2014/5* (Sheldrick, 2015*a* ▸), *SHELXL* (Sheldrick, 2015*b* ▸), and *OLEX2* (Dolomanov *et al.*, 2009 ▸).

## supplementary crystallographic information

### catena-Poly[[chloridolithium(I)]-µ-(dimethyl sulfoxide)-κ2O:O-[chloridolithium(I)]-di-µ-(dimethyl sulfoxide)-κ4O:O] (Monoclinic) Crystal data

**Table:**

[Li_2_Cl_2_(C_2_H_6_OS)_3_]	*F*(000) = 664
*M**_r_* = 319.16	*D*_x_ = 1.416 Mg m^−^^3^
Monoclinic, *C*2/*c*	Cu *K*α radiation, λ = 1.54178 Å
*a* = 19.2841 (17) Å	Cell parameters from 4022 reflections
*b* = 7.6436 (7) Å	θ = 5.2–71.2°
*c* = 11.5335 (10) Å	µ = 7.72 mm^−^^1^
β = 118.315 (5)°	*T* = 100 K
*V* = 1496.6 (2) Å^3^	Block, colourless
*Z* = 4	0.07 × 0.07 × 0.05 mm

### catena-Poly[[chloridolithium(I)]-µ-(dimethyl sulfoxide)-κ2O:O-[chloridolithium(I)]-di-µ-(dimethyl sulfoxide)-κ4O:O] (Monoclinic) Data collection

**Table:**

Bruker APEXII CCD diffractometer	1166 reflections with *I* > 2σ(*I*)
φ and ω scans	*R*_int_ = 0.064
Absorption correction: multi-scan (SADABS; Krause *et al.*, 2015)	θ_max_ = 70.0°, θ_min_ = 5.2°
*T*_min_ = 0.639, *T*_max_ = 0.754	*h* = −22→23
10606 measured reflections	*k* = −9→9
1417 independent reflections	*l* = −14→14

### catena-Poly[[chloridolithium(I)]-µ-(dimethyl sulfoxide)-κ2O:O-[chloridolithium(I)]-di-µ-(dimethyl sulfoxide)-κ4O:O] (Monoclinic) Refinement

**Table:**

Refinement on *F*^2^	68 restraints
Least-squares matrix: full	Hydrogen site location: mixed
*R*[*F*^2^ > 2σ(*F*^2^)] = 0.035	H atoms treated by a mixture of independent and constrained refinement
*wR*(*F*^2^) = 0.081	*w* = 1/[σ^2^(*F*_o_^2^) + (0.0023*P*)^2^ + 6.8363*P*] where *P* = (*F*_o_^2^ + 2*F*_c_^2^)/3
*S* = 1.09	(Δ/σ)_max_ = 0.001
1417 reflections	Δρ_max_ = 0.46 e Å^−^^3^
92 parameters	Δρ_min_ = −0.39 e Å^−^^3^

### catena-Poly[[chloridolithium(I)]-µ-(dimethyl sulfoxide)-κ2O:O-[chloridolithium(I)]-di-µ-(dimethyl sulfoxide)-κ4O:O] (Monoclinic) Special details

**Table:**

Geometry. All esds (except the esd in the dihedral angle between two l.s. planes) are estimated using the full covariance matrix. The cell esds are taken into account individually in the estimation of esds in distances, angles and torsion angles; correlations between esds in cell parameters are only used when they are defined by crystal symmetry. An approximate (isotropic) treatment of cell esds is used for estimating esds involving l.s. planes.

### catena-Poly[[chloridolithium(I)]-µ-(dimethyl sulfoxide)-κ2O:O-[chloridolithium(I)]-di-µ-(dimethyl sulfoxide)-κ4O:O] (Monoclinic) Fractional atomic coordinates and isotropic or equivalent isotropic displacement parameters (Å^2^)

**Table:**

	*x*	*y*	*z*	*U*_iso_*/*U*_eq_	Occ. (<1)
Li1	0.4831 (3)	0.5661 (7)	0.3715 (4)	0.0215 (10)	
Cl1	0.39011 (4)	0.78167 (9)	0.26503 (6)	0.02346 (19)	
O1	0.500000	0.4171 (4)	0.250000	0.0231 (6)	
S1	0.46168 (7)	0.23074 (17)	0.24491 (12)	0.0150 (3)	0.5
C1	0.46522 (18)	0.1205 (4)	0.1177 (3)	0.0227 (6)	
H1A	0.463 (2)	−0.006 (2)	0.125 (4)	0.052 (12)*	
H1B	0.439 (2)	0.178 (5)	0.033 (2)	0.052 (12)*	
H1C	0.519 (2)	0.121 (12)	0.129 (9)	0.21 (4)*	
O2	0.57073 (10)	0.5856 (3)	0.55161 (17)	0.0184 (4)	
S2	0.65940 (4)	0.60785 (9)	0.63468 (6)	0.01629 (18)	
C2	0.68091 (16)	0.8173 (4)	0.5918 (3)	0.0207 (6)	
H2A	0.659005	0.824918	0.496007	0.031*	
H2B	0.738110	0.833830	0.633975	0.031*	
H2C	0.657431	0.908521	0.621951	0.031*	
C3	0.70219 (17)	0.4751 (4)	0.5580 (3)	0.0229 (6)	
H3A	0.694545	0.351352	0.571155	0.034*	
H3B	0.758674	0.500205	0.597297	0.034*	
H3C	0.676854	0.500799	0.463501	0.034*	

### catena-Poly[[chloridolithium(I)]-µ-(dimethyl sulfoxide)-κ2O:O-[chloridolithium(I)]-di-µ-(dimethyl sulfoxide)-κ4O:O] (Monoclinic) Atomic displacement parameters (Å^2^)

**Table:**

	*U*^11^	*U*^22^	*U*^33^	*U*^12^	*U*^13^	*U*^23^
Li1	0.017 (2)	0.032 (3)	0.014 (2)	−0.001 (2)	0.0064 (18)	−0.0028 (19)
Cl1	0.0195 (3)	0.0272 (4)	0.0174 (3)	−0.0028 (3)	0.0037 (3)	0.0043 (3)
O1	0.0408 (17)	0.0139 (13)	0.0175 (14)	0.000	0.0162 (13)	0.000
S1	0.0136 (6)	0.0169 (6)	0.0141 (6)	−0.0003 (5)	0.0063 (5)	−0.0001 (5)
C1	0.0275 (16)	0.0185 (15)	0.0152 (14)	−0.0019 (13)	0.0045 (13)	−0.0008 (11)
O2	0.0111 (9)	0.0284 (11)	0.0139 (9)	−0.0011 (8)	0.0046 (7)	0.0003 (8)
S2	0.0125 (3)	0.0213 (4)	0.0138 (3)	−0.0014 (3)	0.0052 (3)	0.0013 (3)
C2	0.0189 (14)	0.0214 (15)	0.0198 (14)	−0.0024 (11)	0.0074 (12)	0.0001 (11)
C3	0.0202 (14)	0.0260 (16)	0.0238 (15)	−0.0008 (12)	0.0114 (12)	−0.0019 (12)

### catena-Poly[[chloridolithium(I)]-µ-(dimethyl sulfoxide)-κ2O:O-[chloridolithium(I)]-di-µ-(dimethyl sulfoxide)-κ4O:O] (Monoclinic) Geometric parameters (Å, º)

**Table:**

Li1—Li1^i^	3.162 (9)	C1—H1A	0.972 (14)
Li1—Li1^ii^	2.899 (9)	C1—H1B	0.967 (14)
Li1—Cl1	2.313 (5)	C1—H1C	0.974 (15)
Li1—O1	1.949 (5)	O2—S2	1.5219 (18)
Li1—S1	2.881 (5)	S2—C2	1.782 (3)
Li1—O2^ii^	2.021 (5)	S2—C3	1.785 (3)
Li1—O2	1.966 (5)	C2—H2A	0.9800
Li1—S2^ii^	3.024 (5)	C2—H2B	0.9800
O1—S1	1.593 (3)	C2—H2C	0.9800
O1—S1^i^	1.593 (3)	C3—H3A	0.9800
S1—S1^i^	1.426 (2)	C3—H3B	0.9800
S1—C1	1.721 (3)	C3—H3C	0.9800
S1—C1^i^	1.758 (3)		
			
Li1^ii^—Li1—Li1^i^	149.9 (3)	S1^i^—S1—O1	63.41 (6)
Li1^ii^—Li1—S2^ii^	68.36 (17)	S1^i^—S1—C1^i^	64.48 (13)
Cl1—Li1—Li1^ii^	122.2 (3)	S1^i^—S1—C1	67.14 (13)
Cl1—Li1—Li1^i^	87.91 (16)	C1^i^—S1—Li1	96.24 (14)
Cl1—Li1—S1	118.43 (18)	C1—S1—Li1	144.41 (15)
Cl1—Li1—S2^ii^	80.40 (13)	C1—S1—C1^i^	101.25 (18)
O1—Li1—Li1^ii^	119.9 (3)	S1—C1—S1^i^	48.38 (11)
O1—Li1—Li1^i^	35.76 (16)	S1^i^—C1—H1A	117 (2)
O1—Li1—Cl1	112.8 (2)	S1—C1—H1A	113 (2)
O1—Li1—S1	31.64 (11)	S1—C1—H1B	115 (2)
O1—Li1—O2	116.8 (2)	S1^i^—C1—H1B	120 (2)
O1—Li1—O2^ii^	106.0 (3)	S1—C1—H1C	111 (5)
O1—Li1—S2^ii^	100.72 (19)	S1^i^—C1—H1C	62 (5)
S1—Li1—Li1^i^	65.90 (10)	H1A—C1—H1B	121 (3)
S1—Li1—Li1^ii^	96.7 (2)	H1A—C1—H1C	95 (5)
S1—Li1—S2^ii^	71.72 (12)	H1B—C1—H1C	98 (5)
O2^ii^—Li1—Li1^ii^	42.59 (14)	Li1—O2—Li1^ii^	93.3 (2)
O2—Li1—Li1^i^	120.1 (3)	S2—O2—Li1^ii^	116.46 (16)
O2—Li1—Li1^ii^	44.10 (13)	S2—O2—Li1	145.04 (17)
O2^ii^—Li1—Li1^i^	138.89 (18)	O2—S2—Li1^ii^	36.75 (11)
O2^ii^—Li1—Cl1	102.2 (2)	O2—S2—C2	105.41 (12)
O2—Li1—Cl1	124.6 (2)	O2—S2—C3	105.66 (12)
O2^ii^—Li1—S1	74.44 (17)	C2—S2—Li1^ii^	135.83 (14)
O2—Li1—S1	116.6 (2)	C2—S2—C3	98.65 (14)
O2—Li1—O2^ii^	86.7 (2)	C3—S2—Li1^ii^	111.54 (14)
O2^ii^—Li1—S2^ii^	26.78 (8)	S2—C2—H2A	109.5
O2—Li1—S2^ii^	111.96 (19)	S2—C2—H2B	109.5
S2^ii^—Li1—Li1^i^	123.1 (2)	S2—C2—H2C	109.5
Li1^i^—O1—Li1	108.5 (3)	H2A—C2—H2B	109.5
S1—O1—Li1^i^	136.79 (16)	H2A—C2—H2C	109.5
S1^i^—O1—Li1^i^	108.45 (16)	H2B—C2—H2C	109.5
S1—O1—Li1	108.45 (16)	S2—C3—H3A	109.5
S1^i^—O1—Li1	136.79 (16)	S2—C3—H3B	109.5
S1—O1—S1^i^	53.18 (13)	S2—C3—H3C	109.5
O1—S1—Li1	39.92 (10)	H3A—C3—H3B	109.5
O1—S1—C1^i^	103.64 (12)	H3A—C3—H3C	109.5
O1—S1—C1	105.29 (13)	H3B—C3—H3C	109.5
S1^i^—S1—Li1	93.68 (11)		
			
Li1^i^—O1—S1—Li1	−147.5 (2)	Li1—O2—S2—C2	63.5 (4)
Li1^i^—O1—S1—S1^i^	77.3 (2)	Li1^ii^—O2—S2—C2	−151.0 (2)
Li1—O1—S1—S1^i^	−135.24 (17)	Li1^ii^—O2—S2—C3	105.2 (2)
Li1^i^—O1—S1—C1^i^	129.2 (2)	Li1—O2—S2—C3	−40.4 (4)
Li1—O1—S1—C1	170.72 (19)	O1—S1—C1—S1^i^	51.77 (9)
Li1^i^—O1—S1—C1	23.3 (3)	S1^i^—O1—S1—Li1	135.24 (17)
Li1—O1—S1—C1^i^	−83.35 (19)	S1^i^—O1—S1—C1^i^	51.89 (13)
Li1—S1—C1—S1^i^	62.0 (2)	S1^i^—O1—S1—C1	−54.04 (13)
Li1—O2—S2—Li1^ii^	−145.5 (3)	C1^i^—S1—C1—S1^i^	−55.91 (15)

Symmetry codes: (i) −*x*+1, *y*, −*z*+1/2; (ii) −*x*+1, −*y*+1, −*z*+1.

### catena-Poly[[chloridolithium(I)]-µ-(dimethyl sulfoxide)-κ2O:O-[chloridolithium(I)]-di-µ-(dimethyl sulfoxide)-κ4O:O] (Monoclinic) Hydrogen-bond geometry (Å, º)

**Table:**

*D*—H···*A*	*D*—H	H···*A*	*D*···*A*	*D*—H···*A*
C1—H1*A*···Cl1^iii^	0.97 (1)	2.98 (4)	3.569 (3)	120 (3)
C1—H1*B*···Cl1^iv^	0.97 (1)	2.79 (2)	3.690 (3)	156 (3)
C2—H2*A*···Cl1^i^	0.98	2.71	3.680 (3)	169
C2—H2*B*···Cl1^v^	0.98	2.73	3.632 (3)	153
C3—H3*B*···Cl1^v^	0.98	2.88	3.752 (3)	149

Symmetry codes: (i) −*x*+1, *y*, −*z*+1/2; (iii) −*x*+1, *y*−1, −*z*+1/2; (iv) *x*, −*y*+1, *z*−1/2; (v) *x*+1/2, −*y*+3/2, *z*+1/2.

### catena-Poly[lithium(I)-µ-chlorido-µ-(dimethyl sulfoxide)-κ2O:O] (Tetragonal) Crystal data

**Table:**

[LiCl(C_2_H_6_OS)]	*D*_x_ = 1.451 Mg m^−^^3^
*M**_r_* = 120.52	Cu *K*α radiation, λ = 1.54178 Å
Tetragonal, *I*4_1_/*a*	Cell parameters from 6535 reflections
*a* = 14.2411 (14) Å	θ = 4.4–72.3°
*c* = 10.8809 (16) Å	µ = 8.49 mm^−^^1^
*V* = 2206.7 (5) Å^3^	*T* = 100 K
*Z* = 16	Octahedron, clear colourless
*F*(000) = 992	0.4 × 0.4 × 0.4 mm

### catena-Poly[lithium(I)-µ-chlorido-µ-(dimethyl sulfoxide)-κ2O:O] (Tetragonal) Data collection

**Table:**

Bruker APEXII CCD diffractometer	1030 reflections with *I* > 2σ(*I*)
φ and ω scans	*R*_int_ = 0.047
Absorption correction: multi-scan (SADABS; Krause *et al.*, 2015)	θ_max_ = 72.3°, θ_min_ = 5.1°
*T*_min_ = 0.513, *T*_max_ = 0.754	*h* = −17→16
8174 measured reflections	*k* = −16→17
1072 independent reflections	*l* = −13→13

### catena-Poly[lithium(I)-µ-chlorido-µ-(dimethyl sulfoxide)-κ2O:O] (Tetragonal) Refinement

**Table:**

Refinement on *F*^2^	0 restraints
Least-squares matrix: full	Hydrogen site location: difference Fourier map
*R*[*F*^2^ > 2σ(*F*^2^)] = 0.028	All H-atom parameters refined
*wR*(*F*^2^) = 0.073	*w* = 1/[σ^2^(*F*_o_^2^) + (0.0416*P*)^2^ + 1.762*P*] where *P* = (*F*_o_^2^ + 2*F*_c_^2^)/3
*S* = 1.07	(Δ/σ)_max_ = 0.001
1072 reflections	Δρ_max_ = 0.32 e Å^−^^3^
79 parameters	Δρ_min_ = −0.37 e Å^−^^3^

### catena-Poly[lithium(I)-µ-chlorido-µ-(dimethyl sulfoxide)-κ2O:O] (Tetragonal) Special details

**Table:**

Geometry. All esds (except the esd in the dihedral angle between two l.s. planes) are estimated using the full covariance matrix. The cell esds are taken into account individually in the estimation of esds in distances, angles and torsion angles; correlations between esds in cell parameters are only used when they are defined by crystal symmetry. An approximate (isotropic) treatment of cell esds is used for estimating esds involving l.s. planes.

### catena-Poly[lithium(I)-µ-chlorido-µ-(dimethyl sulfoxide)-κ2O:O] (Tetragonal) Fractional atomic coordinates and isotropic or equivalent isotropic displacement parameters (Å^2^)

**Table:**

	*x*	*y*	*z*	*U*_iso_*/*U*_eq_	
Cl1	0.81811 (3)	0.36163 (2)	0.89755 (3)	0.02090 (17)	
S1	0.58855 (3)	0.44534 (2)	0.62126 (3)	0.01513 (16)	
O1	0.69499 (7)	0.45632 (8)	0.62414 (8)	0.0182 (3)	
C1	0.56163 (11)	0.37206 (12)	0.74923 (14)	0.0238 (3)	
H1A	0.6017 (15)	0.3177 (14)	0.7468 (19)	0.035 (5)*	
H1B	0.4949 (14)	0.3561 (14)	0.7438 (18)	0.032 (5)*	
H1C	0.5707 (14)	0.4087 (14)	0.822 (2)	0.030 (5)*	
C2	0.56716 (10)	0.36370 (11)	0.50047 (14)	0.0194 (3)	
H2A	0.6049 (14)	0.3099 (14)	0.5150 (18)	0.030 (5)*	
H2B	0.5842 (13)	0.3952 (14)	0.425 (2)	0.029 (5)*	
H2C	0.5011 (14)	0.3503 (14)	0.5012 (18)	0.030 (5)*	
Li1	0.77950 (17)	0.48024 (16)	0.7596 (2)	0.0185 (5)	

### catena-Poly[lithium(I)-µ-chlorido-µ-(dimethyl sulfoxide)-κ2O:O] (Tetragonal) Atomic displacement parameters (Å^2^)

**Table:**

	*U*^11^	*U*^22^	*U*^33^	*U*^12^	*U*^13^	*U*^23^
Cl1	0.0232 (2)	0.0197 (2)	0.0198 (2)	0.00734 (14)	0.00006 (13)	0.00281 (13)
S1	0.0163 (2)	0.0145 (2)	0.0145 (2)	−0.00053 (13)	0.00054 (12)	0.00014 (11)
O1	0.0173 (6)	0.0243 (6)	0.0128 (5)	−0.0070 (4)	−0.0005 (3)	0.0004 (4)
C1	0.0222 (8)	0.0307 (9)	0.0184 (8)	−0.0065 (7)	0.0026 (6)	0.0052 (6)
C2	0.0176 (7)	0.0212 (7)	0.0193 (7)	−0.0011 (6)	−0.0023 (5)	−0.0025 (6)
Li1	0.0216 (12)	0.0206 (12)	0.0133 (10)	0.0025 (10)	−0.0006 (9)	−0.0006 (9)

### catena-Poly[lithium(I)-µ-chlorido-µ-(dimethyl sulfoxide)-κ2O:O] (Tetragonal) Geometric parameters (Å, º)

**Table:**

Cl1—Li1	2.326 (2)	C1—H1B	0.98 (2)
Cl1—Li1^i^	2.336 (2)	C1—H1C	0.96 (2)
S1—O1	1.5241 (11)	C2—H2A	0.95 (2)
S1—C1	1.7819 (15)	C2—H2B	0.97 (2)
S1—C2	1.7810 (15)	C2—H2C	0.96 (2)
O1—Li1^ii^	1.943 (3)	Li1—Li1^i^	2.8126 (10)
O1—Li1	1.933 (3)	Li1—Li1^ii^	2.8127 (10)
C1—H1A	0.96 (2)		
			
Li1—Cl1—Li1^i^	74.22 (7)	H2A—C2—H2C	113.1 (18)
O1—S1—C1	104.95 (7)	H2B—C2—H2C	110.1 (16)
O1—S1—C2	104.61 (6)	Cl1—Li1—Cl1^ii^	124.69 (11)
C2—S1—C1	99.06 (8)	Cl1^ii^—Li1—Li1^i^	134.17 (10)
S1—O1—Li1	130.77 (9)	Cl1—Li1—Li1^ii^	144.79 (11)
S1—O1—Li1^ii^	125.94 (9)	Cl1^ii^—Li1—Li1^ii^	52.72 (8)
Li1—O1—Li1^ii^	93.06 (8)	Cl1—Li1—Li1^i^	53.05 (7)
S1—C1—H1A	108.8 (12)	O1^i^—Li1—Cl1	96.36 (10)
S1—C1—H1B	107.3 (12)	O1^i^—Li1—Cl1^ii^	113.41 (11)
S1—C1—H1C	107.3 (12)	O1—Li1—Cl1^ii^	96.30 (10)
H1A—C1—H1B	112.8 (18)	O1—Li1—Cl1	120.75 (12)
H1A—C1—H1C	112.5 (17)	O1—Li1—O1^i^	104.58 (12)
H1B—C1—H1C	107.9 (16)	O1—Li1—Li1^i^	124.99 (13)
S1—C2—H2A	107.9 (12)	O1^i^—Li1—Li1^i^	43.34 (7)
S1—C2—H2B	106.4 (12)	O1^i^—Li1—Li1^ii^	117.22 (12)
S1—C2—H2C	107.0 (12)	O1—Li1—Li1^ii^	43.60 (6)
H2A—C2—H2B	111.9 (16)	Li1^i^—Li1—Li1^ii^	159.29 (10)
			
C1—S1—O1—Li1^ii^	−179.33 (12)	C2—S1—O1—Li1^ii^	76.90 (13)
C1—S1—O1—Li1	−43.92 (15)	C2—S1—O1—Li1	−147.69 (13)

Symmetry codes: (i) *y*+1/4, −*x*+5/4, *z*+1/4; (ii) −*y*+5/4, *x*−1/4, *z*−1/4.

### catena-Poly[lithium(I)-µ-chlorido-µ-(dimethyl sulfoxide)-κ2O:O] (Tetragonal) Hydrogen-bond geometry (Å, º)

**Table:**

*D*—H···*A*	*D*—H	H···*A*	*D*···*A*	*D*—H···*A*
C1—H1*B*···Cl1^iii^	0.98 (2)	2.95 (2)	3.821 (2)	148 (2)
C2—H2*A*···Cl1^iv^	0.95 (2)	2.84 (2)	3.768 (2)	165 (2)
C2—H2*C*···Cl1^iii^	0.96 (2)	2.83 (2)	3.716 (2)	153 (2)

Symmetry codes: (iii) *x*−1/2, *y*, −*z*+3/2; (iv) −*x*+3/2, −*y*+1/2, −*z*+3/2.
